# Genomic Differences and Distinct *TP53* Mutation Site‐Linked Chemosensitivity in Early‐ and Late‐Onset Gastric Cancer

**DOI:** 10.1002/cam4.70793

**Published:** 2025-04-18

**Authors:** Tomohiro Kamio, Yoshiyasu Kono, Kensuke Hirosuna, Toshiki Ozato, Hideki Yamamoto, Akira Hirasawa, Daisuke Ennishi, Shuta Tomida, Shinichi Toyooka, Motoyuki Otsuka

**Affiliations:** ^1^ Department of Gastroenterology and Hepatology, Faculty of Medicine, Dentistry and Pharmaceutical Sciences Okayama University Okayama Japan; ^2^ Department of Regenerative Science Okayama University Graduate School of Medicine, Dentistry and Pharmaceutical Sciences Okayama Japan; ^3^ Department of Gastroenterology Okayama University Hospital Okayama Japan; ^4^ Department of Clinical Genomic Medicine Okayama University Hospital Okayama Japan; ^5^ Center for Comprehensive Genomic Medicine Okayama University Hospital Okayama Japan; ^6^ Department of General Thoracic Surgery, Breast and Endocrinological Surgery Faculty of Medicine, Dentistry and Pharmaceutical Sciences Okayama Japan

**Keywords:** comprehensive genomic profiling, early‐onset gastric cancer, oxaliplatin, *TP53*

## Abstract

**Background:**

Gastric cancer (GC) in younger patients often exhibits aggressive behavior and a poorer prognosis than that in older patients. Although the clinical differences may stem from oncogenic gene variations, it is unclear whether genetic differences exist between these groups. This study compared the genetic profiles of early‐ and late‐onset GC and evaluated their impact on treatment outcomes.

**Methods:**

We analyzed genetic data from 1284 patients with GC in the Japanese nationwide Center for Cancer Genomics and Advanced Therapeutics (C‐CAT) database, comparing early‐onset (≤ 39 years; *n* = 143) and late‐onset (≥ 65 years; *n* = 1141) groups. The influence of TP53 mutations on the time to treatment failure (TTF) with platinum‐based chemotherapy and the sensitivity of cancer cells with different TP53 mutation sites to oxaliplatin were assessed in vitro.

**Results:**

Early‐ and late‐onset GC showed distinct genetic profiles, with fewer neoantigen‐associated genetic changes observed in early‐onset cases. In particular, TP53 has distinct mutation sites; R175H and R273 mutations are more frequent in early‐ and late‐onset GC, respectively. The R175H mutation showed higher sensitivity to oxaliplatin in vitro, consistent with the longer TTF in early‐onset patients (17.3 vs. 7.0 months, *p* = 0.013) when focusing on the patients with TP53 mutations.

**Conclusion:**

Genomic differences, particularly in TP53 mutation sites, between early‐ and late‐onset GC support the need for age‐specific treatment strategies.

## Graphical abstract

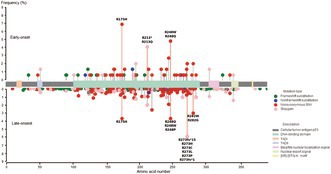

**Abbreviations**
C‐CAT: Center for Cancer Genomics and Advanced Therapeutics
CGP: comprehensive genomic profiling
CI: confidence interval
CNV: copy number variants
DMEM: Dulbecco's modified Eagle's medium
ECOG PS: Eastern Cooperative Oncology Group Performance Status
FBS: fetal bovine serum
GC: gastric cancer
HER2: human epidermal growth factor receptor 2
HRP: Horseradish peroxidase
MSI: microsatellite instability
nanoLuc: nano‐luciferase
Nivo: nivolumab
OS: overall survival
PBS: phosphate‐buffered saline
PVDF: polyvinylidene difluoride
QC: quality check
RE: response element
RPMI: Roswell Park Memorial Institute
SD: standard deviation
SNV: single nucleotide variants
Tmab: trastuzumab
TMB: tumor mutation burden
TTF: time to treatment failure
VUS: variant of unknown significance

## 1 Introduction

Gastric cancer (GC) remains a major contributor to cancer‐related mortality worldwide [1]. Despite significant advancements in treatment modalities, the outcomes for patients with GC, especially those in advanced stages, remain dismal [2, 3]. The disease affects diverse age groups, with notable differences in the clinical characteristics between early‐ and late‐onset GC. Early‐onset GC often demonstrates a more aggressive behavior and poorer prognosis than late‐onset GC [4]. The 5‐year relative survival rate for late‐onset GC is reported to be 28.9%, compared to 28.1% for early‐onset GC [5]. Factors contributing to poor prognosis in younger patients include undifferentiated histology, unresectable tumors, lymphovascular invasion, and advanced disease stage at diagnosis [6, 7].

Genetic variations are believed to play critical roles in the clinical disparities among GCs [8]. However, the precise genetic mechanisms underlying the clinical differences between early‐ and late‐onset GC are not fully understood. Current research on the genomic disparities between these two subsets is limited, leading to significant uncertainties. Key gaps include identifying specific genetic mutations associated with each subtype, elucidating the pathways governing disease progression, and understanding how genetic diversity influences treatment response.

In this study, we aimed to address these knowledge gaps using a comprehensive nationwide genetic database to compare the genetic profiles of early‐ and late‐onset GC and examine their associations with treatment outcomes.

## 2 Materials and Methods

### 2.1 Study Design

Patient data included in this retrospective observational study were retrieved from the Center for Cancer Genomics and Advanced Therapeutics (C‐CAT) utilization portal, a comprehensive nationwide database for cancer genomics in Japan. Data from patients diagnosed with advanced or recurrent GC registered in the C‐CAT portal between June 2019 and February 2024 were used for analyses. This study adhered to the Declaration of Helsinki and was approved by the Ethics Committee of our institution (approval number: 2111‐047) and the Review Board of C‐CAT (C‐CAT Control Number: CDU2022‐012E02). All patients enrolled in the Portal have given written informed consent permitting the secondary use of their clinical data and CGP results for research purposes. Subjects were stratified into two groups according to the age at which GC was detected and registered: the early‐onset (age, ≤ 39 years; *n* = 143) and late‐onset (age, ≥ 65 years; *n* = 1141) groups. The cutoff age for early‐onset and late‐onset was determined with reference to previous reports [9, 10, 11].

### 2.2 Clinical Data Collection

Patient characteristics and treatment‐related data, including age, sex, Eastern Cooperative Oncology Group Performance Status (ECOG PS), smoking history, alcohol consumption, primary site, main histological type, human epidermal growth factor receptor 2 (HER2) status, type of comprehensive genomic profiling (CGP) testing, tissue sampling area, and chemotherapy regimen with treatment lines, were collected using standard data collection procedures.

### 2.3 Comprehensive Genomic Profiling Testing

CGP assays were performed using the Foundation One CDx (Foundation Medicine), the OncoGuide NCC Oncopanel System (Sysmex Corporation), and the GeneMine TOP (Konica Minolta Inc.) to analyze formalin‐fixed paraffin‐embedded (FFPE) tumor tissue samples. Foundation One Liquid CDx (Foundation Medicine) and Guardant 360 CDx (Guardant Health Incorporated) were used for examining the circulating tumor DNA (ctDNA).

Before submitting the tissue samples for panel testing, a quality check (QC) of the nucleic acids, including nucleic acid yield, concentration, and fragmentation, was performed. If the QC results were satisfactory, tissue‐based panel testing was prioritized. Liquid‐based panel testing was conducted in case of quality issues or insufficient nucleic acid yields.

### 2.4 Bioinformatic Analysis

The clinical implications of the detected gene variations were classified as oncogenic, pathogenic, likely oncogenic, likely pathogenic, benign, likely benign, inconclusive, variant of unknown significance (VUS), or unknown according to the clinical annotation of C‐CAT. This classification is based on the Cancer Knowledge Database constructed using C‐CAT, which compiles information on gene mutations, drugs, and clinical trials from public genomic medicine‐related databases available worldwide [12]. ANNOVAR: 20210202 was used to organize the annotation process [13].

### 2.5 Outcomes

We compared CGP and clinicopathological characteristics between the early‐ and late‐onset groups. To visualize the differences between the two groups, an oncoprint was generated using the comut software (https://github.com/vanallenlab/comut). The dataset was processed to include genomic alterations, such as single nucleotide variants (SNVs), copy number variants (CNVs), microsatellite instability (MSI), and tumor mutation burden (TMB). For the comparison of CGP in the early‐ and late‐onset groups, all variants were considered regardless of their significance.

Based on the CGP differences between the early‐ and late‐onset groups, we subsequently examined the influence of the predominant variant on the therapeutic effectiveness of platinum doublet chemotherapy as the first‐line regimen, as indicated by the time to treatment failure (TTF), across both study groups. Only patients who received regimens for unresectable cancer were included, after excluding those who received neoadjuvant and postoperative chemotherapy. Only the variants classified as oncogenic, pathogenic, likely oncogenic, or likely pathogenic were included in the analyses. The lollipop plot was generated to illustrate the distribution of genetic mutations across the genome. The genomic positions of the mutations are plotted on the *x*‐axis, and the frequency or significance of the mutations is plotted on the *y*‐axis. We compared the distribution of predominant variants and TTF outcomes.

### 2.6 Cell Lines

Human GC cell lines AGS (wild‐type p53), KATO III (p53 null) cells, and 293T cells were purchased from the American Type Culture Collection (ATCC Manassas, VA, USA). AGS and 293T cells were cultured in Dulbecco's modified Eagle's medium (DMEM) high glucose (Thermo Fisher Scientific, Waltham, MA, USA) and 10% fetal bovine serum (FBS). KATO III cells were cultured in Roswell Park Memorial Institute (RPMI) 1640 medium (Thermo Fisher Scientific) supplemented with 10% FBS. All cells were cultured at 37°C under 20% O_2_ and 5% CO_2_. All cell lines were confirmed as mycoplasma‐free and authenticated by STR profiling.

### 2.7 Plasmids

To overexpress wildtype p53 and p53 mutants fused with N‐terminal HiBiT tag sequences (VSGWRLFKKIS) lentiviral plasmids were constructed under the EF1α promoter (VectorBuilder, Chicago, IL). R175H was used as a representative p53 mutant in the early‐onset GC group, and R273H and R273fs*1 were used as representative p53 mutants in the late‐onset GC group. For reporter assays to determine TP53 function, a vector containing two copies of a p53 response element (p53 RE) that drives transcription of the luciferase reporter gene (p53RE‐nanoLuc) was constructed (Promega, Madison, WI, USA). For normalization, an internal control, pGL4.54 plasmid‐expressing firefly luciferase under the control of the TK promoter (Promega) was used.

### 2.8 Transfection and Lentiviral Transduction

Transient transfections were performed using Effectene Transfection Reagent (Qiagen, Hilden, Germany). p53RE‐nanoLuc and various types of p53 expressing plasmids were cotransfected with a control plasmid, pGL4.54, to normalize NanoLuc activity. To generate polyclonal cells with various types of p53 stable expression, a Lentivirus Packaging System (System Biosciences, Palo Alto, CA, USA) was used according to the manufacturer's instructions. Briefly, 1.0 μg of various types of p53 constructs and 5.0 μg of pPACKH1 packaging plasmid mix were transfected into 293T cells. After 24 h, the collected culture media were mixed with one‐fifth the volume of PEG‐it Reagent (System Biosciences) and incubated overnight at 4°C to concentrate the viruses. The centrifuged pellet was resuspended in 1× phosphate‐buffered saline (PBS). The viruses were transduced into KATO III cells using Polybrene (Santa Cruz Biotechnology, Dallas, TX, USA), followed by selection with 2 mg/mL puromycin to obtain polyclonal cells that stably expressed various TP53 proteins.

### 2.9 Dual Luciferase Reporter Assay

Luciferase activity was detected using the Nano‐Glo Dual Luciferase Reporter Assay System (Promega), according to the manufacturer's instructions. Briefly, cells in a 24‐well plate were transiently transfected with the indicated plasmids. At 48 h after transfection, One‐Glo EX luciferase assay reagent was added to the culture media, and 20 μL of the supernatant was transferred to the 96‐well white plate, followed by the measurement of firefly luciferase activities using the GloMax Explorer plate reader (Promega). Subsequently, NanoDLR Stop & Glo Reagent was added, and after incubation for 10 min, the supernatant was transferred to a 96‐well white plate, followed by nano‐luciferase activity measurement. The relative nano‐luciferase activity was normalized to the firefly activity.

### 2.10 Western Blotting and HiBiT Blotting

Western blotting was performed as previously described [14]. Briefly, lysate samples were separated on a 10%–20% gradient polyacrylamide gel by sodium dodecyl sulfate‐polyacrylamide gel electrophoresis following electrical transfer to polyvinylidene difluoride (PVDF) membranes. After blocking with 5% dry milk, membranes were probed overnight at 4°C with the appropriate primary antibodies diluted in Immunoshot Reagent 1 (Cosmo Bio Co. Ltd., Tokyo, Japan). Horseradish peroxidase (HRP)‐conjugated corresponding secondary antibodies (GE Healthcare, Little Chalfont, UK) were then applied. Bound antibodies were detected using Immunostar LD reagent (Wako Pure Chemical Industries Ltd., Osaka, Japan). The following antibodies were used: p53 (#9282, 1:1000; Cell Signaling Technology, Danvers, MA); β‐actin (#5125, 1:10,000; Cell Signaling Technology). To detect the HiBiT tag, the Nano‐Glo HiBiT blotting system was used according to the manufacturer's instructions. Briefly, after the transfer, the PVDF membrane was incubated with LgBiT protein overnight at 4°C, followed by detection with Nano‐Glo luciferase assay substrate.

### 2.11 Cell Viability Assay

To determine the sensitivity of KATO III cells to oxaliplatin, cells stably expressing p53 variants were treated with 10 μM of oxaliplatin (Fujifilm Wako, Osaka, Japan) for 3 days. Cell viability was determined using the CellTiter‐Glo 2.0 Cell Viability Assay (Promega), according to the manufacturer's instructions. Briefly, the CellTiter‐Glo reagent was added to the cells in a 48‐well plate, and aliquots of the supernatant were transferred to a 96‐well white plate, followed by luminescence measurements. Relative luminescence was calculated by dividing the values from the treated cells by those from the control cells to determine their sensitivity to oxaliplatin.

### 2.12 Statistical Analyses

For analyses of the results based on the databases, chi‐square tests were used to compare the statistical significance of categorical variables, and Student's *t*‐test was applied for continuous variables. TTF was defined as the time from the start of treatment to treatment discontinuation or death from any cause and was calculated with a 95% confidence interval (CI) using the Kaplan–Meier method and compared between groups using the log‐rank test. All statistical tests were two‐sided, and a *p* value of less than 0.05 was considered statistically significant. Statistical analyses were performed using JMP Pro 17 software (SAS Institute Inc., Cary, NC, USA). For the analyses of in vitro results, variables were reported as mean ± standard deviation (SD). The Welch *t*‐test was used for group comparisons. Statistical analyses were performed using GraphPad Prism 10 software (GraphPad Software, Boston, MA, USA).

## 3 Results

### 3.1 Patient Characteristics

**FIGURE 1:**
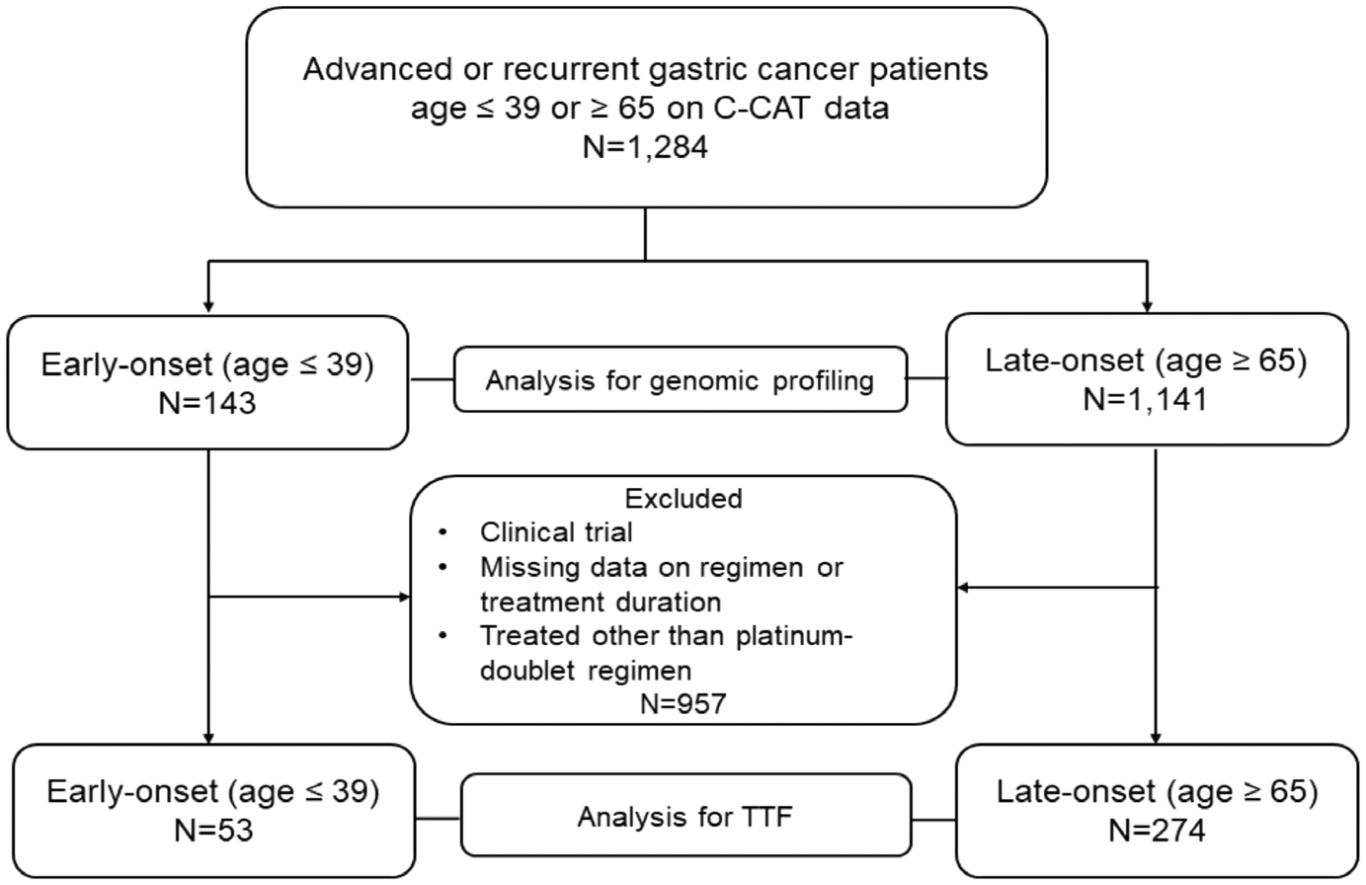
Flow diagram of this study. We first extracted data from 1284 patients diagnosed with advanced or recurrent gastric cancer and registered in the Center for Cancer Genomics and Advanced Therapeutics database (C‐CAT database) between June 2019 and February 2024 (early‐onset group [age, ≤ 39 years; *n* = 143] and late‐onset group [age, ≥ 65 years; *n* = 1141]). Genomic profiling was analyzed for the entire cohort. Next, to investigate the therapeutic effects, we analyzed the time to treatment failure (TTF) in patients treated with a platinum‐doublet regimen as first‐line therapy. The exclusion criteria were clinical trials, cases with missing data on survival or treatment details, and cases that received treatment other than the platinum doublet regimen (*n* = 957). Finally, for TTF analysis, 53 and 274 cases in the early‐ and late‐onset groups, respectively, were extracted. C‐CAT, Center for Cancer Genomics and Advanced Therapeutics; *N*, number; TTF, time to treatment failure.

A total of 1284 cases with advanced or recurrent GC were included in this study, categorized into the early‐onset group (*n* = 143; age ≤ 39 years) and late‐onset group (*n* = 1141; age ≥ 65 years) TTF for platinum‐based chemotherapy was evaluated in 53 and 274 patients of the early‐onset and late‐onset groups, respectively, excluding ineligible cases, as illustrated in the eligibility flow diagram (Figure 1).

Table 1 presents the distribution of clinical features of the enrolled patients, including sex, ECOG PS, family history of cancer, smoking status, alcohol consumption, primary cancer site, main histological type, HER2 score, CGP testing, and tissue sampling area (Table 1). Notably, the early‐onset group had a higher proportion of female patients (*p* < 0.001). The late‐onset group exhibited higher rates of smoking (*p* < 0.001) and alcohol consumption (*p* = 0.0013). Significant differences in histological types were observed (*p* < 0.001), with diffuse and special types more prevalent in the early‐onset group and intestinal types more common in the late‐onset group. Tissue sampling areas also differed significantly (*p* = 0.0003), with 95 primary and 33 metastatic samples in the early‐onset group compared to 857 primary and 128 metastatic samples in the late‐onset group. Regarding the types of first‐line chemotherapy regimens, fluorouracil plus platinum therapy was the most common in both groups, while fluorouracil plus platinum therapy combined with nivolumab (Nivo) was significantly more frequent in the early‐onset group compared to the late‐onset group (13% vs. 4.4%). No significant differences were found in the other factors.

**TABLE 1:** Clinical characteristics.

	Early onset	Late onset	*p*
	*n* = 143	*n* = 1141	
Sex, *n* (male/female)	65/78	835/306	< 0.001
ECOG PS 0–1, *n* (%)	136 (94)	1050 (95)	0.24
Family history of cancer *n* (%)	104 (74)	757 (68)	0.23
Smoking, *n* (%)	34 (24)	661 (60)	< 0.001
Alcohol, *n* (%)	9 (7.0)	190 (18)	0.0013
Primary site, *n* (EGJ/stomach)	15/128	102/1039	0.54
Main histological type, *n* (%)			
Diffuse	66 (46)	286 (25)	< 0.001
Intestinal	75 (52)	850 (74)	
Special	2 (1.4)	5 (0.44)	
HER2 score, *n* (0/1+/2+/3+)	83/21/8/19	546/179/136/133	0.073
Genomic profiling test			
F1CDx	110 (77)	866 (76)	0.43
NOP	18 (13)	111 (9.7)	
F1L	15 (10)	146 (13)	
Guardant 360	0	10 (0.88)	
TOP	0	8 (0.70)	
Tissue sampling area, *n* (primary/metastatic)	95/33	857/128	0.0003
Types of first‐line treatment regimens, *n* (%)			
Fluoropyrimidine + platinum	28 (20)	200 (17.5)	0.0025
Nivo + fluoropyrimidine + platinum	19 (13)	50 (4.4)	
Tmab + fluoropyrimidine + platinum	6 (4.2)	50 (4.4)	
Fluoropyrimidine monotherapy	5 (3.5)	60 (5.3)	
Nivo monotherapy	1 (0.7)	7 (0.6)	
Others or unknown	84 (59)	768 (67)	

*Note:* Regarding the types of first‐line treatment regimens, fluoropyrimidine includes S‐1, capecitabine, and 5‐FU, whereas platinum agents include oxaliplatin and cisplatin.

Abbreviations: ECOG‐PS, Eastern Cooperative Oncology Group performance status; EGJ, esophagogastric junction; F1CDx, FoundationOne CDx; F1L, FoundationOne Liquid CDx; Guardant 360, Guardant 360 CDx; HER2, human epidermal growth factor receptor 2; Nivo, nivolumab; NOP, OncoGuide NCC Oncopanel System; Tmab, trastuzumab; TOP, GenMine TOP.

### 3.2 Genetic Variant Landscape in Early‐ and Late‐Onset Groups With GC

**FIGURE 2:**
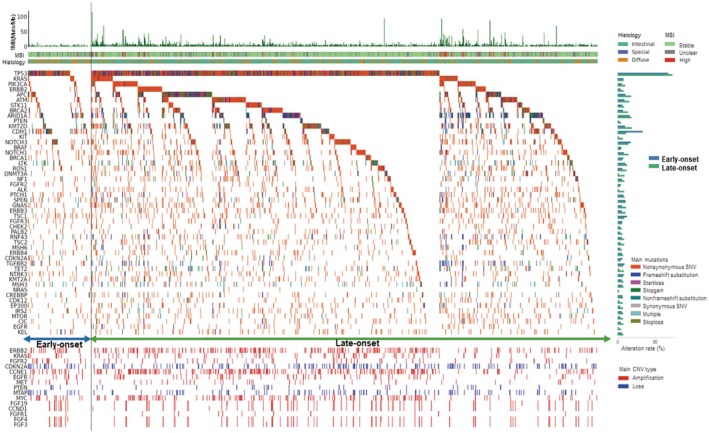
Mutation landscape in the gastric cancer of the 1284 patients. Oncoprints of commonly occurring mutations, including microsatellite instability (MSI) and tumor mutation burden (TMB). The patients were classified into early‐onset (*n* = 143, indicated by the blue arrow) and late‐onset groups (*n* = 1141; indicated by the green arrow). The distribution of single nucleotide variants (SNVs) is shown in the upper part of the panel. Copy number variants (CNVs) are shown in the lower panel. SNVs and CNVs were further distinguished by color‐coding based on the type of mutation. A bar plot of the frequency of each single nucleotide variant (SNV) is shown on the right side of the panel. MSI‐high and TMB‐high are less common in the early‐onset group than in the late‐onset group. *TP53* is the gene that most frequently harbors SNVs. Other highly frequent SNVs were detected in *CDH1, ARID1A, KMT2D, NOTCH3*, and *TSC* genes. The frequency of SNVs in *CDH1* was significantly higher in the early‐onset group than in the late‐onset group (34% vs. 11%, *p* < 0.001). Highly frequent CNVs included *ERBB2*, *FGFR2*, *CDKN2A*, *CCNE1*, *MTAP*, and *MYC*. CNV, copy number variant; MSI, microsatellite instability; SNV, single nucleotide variant; TMB, tumor mutation burden.

Utilizing the information in the C‐CAT database reflecting the nationwide CGP test, the overall genomic alterations, including MSI and TMB status, in the early‐ and late‐onset groups are summarized as follows (Figure 2). Among the SNVs, *TP53* was the most frequently mutated gene in both groups (early‐onset group, 69%; late‐onset group, 74%), although the difference was not significant. Genes with the following frequent variants were identified in the early‐onset group: *CDH1* (34%), *ARID1A* (26%), *NOTCH3* (17%), and *TSC1* (13%). In the late‐onset group, frequent variants were observed in *ARID1A* (34%), *KMT2D* (19%), *APC* (18%), and *ATM* (16%). In addition, among the highly frequent SNVs, significant differences were observed between the early‐ and late‐onset groups for *CDH1* (early‐onset: 34% vs. late‐onset: 11%, *p* < 0.001), *KMT2D* (early‐onset: 20% vs. late‐onset: 33%, *p* < 0.001), *APC* (early‐onset: 11% vs. late‐onset: 18%, *p* = 0.036), and *ATM* (early‐onset: 9.8% vs. late‐onset: 19%, *p* = 0.004). Notably, no cases with MSI‐high were observed in the early‐onset group, whereas 35 cases (3.1%) were observed in the late‐onset group (*p* = 0.027). The median TMB was significantly higher in the late‐onset group than in the early‐onset group (4.7 vs. 2, *p* < 0.001). These results suggest that, while *TP53* mutations are commonly observed, other genetic changes in genes such as *CDH1*, *KMT2D*, *APC*, *ATM*, MSI‐H, and TMB differ between early‐ and late‐onset cases of GC. In particular, early‐onset GC generally has few genetic changes associated with the expression of neoantigens.

### 3.3 Distinct Frequencies of Hotspot Mutation Sites in the *TP53* Gene in GC Between Early‐ and Late‐Onset Groups

**FIGURE 3:**
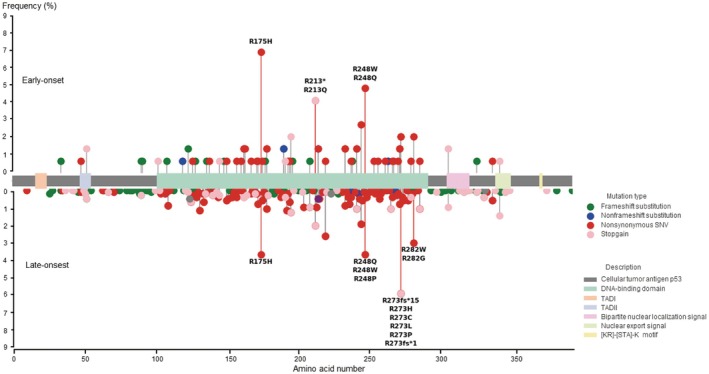
Lollipop plot depicting mutation frequencies of *TP53* in early‐ and late‐onset gastric cancer groups. A lollipop plot illustrating the frequency distribution of *TP53* mutation types around the hot spots in early‐ and late‐onset gastric cancer groups is shown. The *x*‐axis represents the types of variants analyzed, and the *y*‐axis indicates the frequencies for each variant type. Notable differences in frequency distributions were observed for R175H, R213, R273, and R282 between the early‐ and late‐onset groups. SNV, single‐nucleotide variant; TAD, amino‐terminal transactivation domain.

Although significant differences in some genetic variations were observed in GC between the early‐ and late‐onset groups, *TP53* mutations were the most frequent and common in both groups. We further examined the variant types of *TP53* mutations in early‐ and late‐onset groups. Interestingly, the mutation frequencies at hotspot sites of *TP53* varied depending on the hotspot locations in GC between the early‐ and late‐onset groups (Figure 3). R175H, R213*, and R213Q were frequently observed in the early‐onset group, whereas R273fs*15, R273H, R273C, R273L, R273P, R273fr*1, R282W, and R282G were more common in the late‐onset group. These findings demonstrate the intriguing disparities in the prevalence of mutation hotspots within the *TP53* gene in GC between early‐ and late‐onset groups. These variations in hotspot frequencies underscore the distinct mutational landscapes across different age groups in GC.

### 3.4 Differences in TTF With Platinum‐Doublet Regimens for GC According to the Early‐ Or Late‐Onset

**FIGURE 4:**
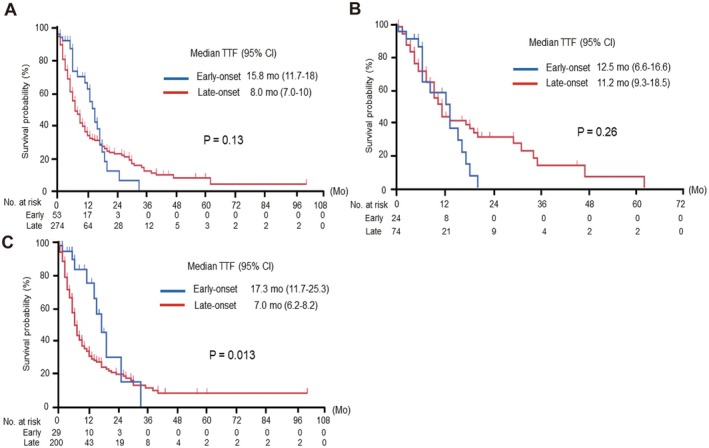
Time to treatment failure in early‐ and late‐onset groups conducting the platinum‐doublet regimen as first‐line chemotherapy. (A) Kaplan–Meier curves of time to treatment failure (TTF) when using the platinum‐doublet regimen in all early‐ and late‐onset groups. The curves are crossing, and median TTFs in early‐ and late‐onset groups are not significantly different (15.8 months vs. 8.0 months, *p* = 0.13). (B) The Kaplan–Meier curves of TTF in patients without the *TP53* mutation (*TP53* wild) group. (C) The Kaplan–Meier curves of TTF in patients with the *TP53* mutation (*TP53* mutant) group. According to the subgroup analyses based on the *TP53* mutation, the crossing of curves has become less noticeable. That indicates median TTF in early‐ and late‐onset groups differs in a *TP53* mutation‐dependent manner. CI, confidence interval; Mo, months, No, number; TTF, time to treatment failure.

Finally, we examined the effect of *TP53* mutations on TTF in patients who received platinum‐doublet regimens as first‐line treatment. Regardless of *TP53* mutation status, the median TTF was worse in the late‐onset group than in the early‐onset group, although the difference was not significant (15.8 vs. 8.0 months, *p* = 0.13). Moreover, it is noteworthy that the Kaplan–Meier curves of TTF in the early‐ and late‐onset groups crossed, indicating that some prognostic factors were present (Figure 4A). In contrast, when subgroup analyses were conducted among the groups without the *TP53* mutation (*TP53* wild‐type) (Figure 4B) and among the groups with the *TP53* mutation (*TP53* mutant) (Figure 4C), the Kaplan–Meier curves were more separated. In other words, the presence or absence of *TP53* mutations reversed the TTF outcomes between the early‐ and late‐onset groups. Additionally, we analyzed the TTF in subgroups of patients receiving platinum doublet therapy combined with either trastuzumab (Figure S1A–C) or Nivo (Figure S1D–F), observing consistent results across each group. These results suggest the differential efficacy of platinum‐based chemotherapeutic agents against GC in younger and older patients harboring *TP53* gene mutations. Considering the observed differences in mutational hotspots of *TP53* between different age groups, this implies that sensitivity to chemotherapy might be determined, at least in part, depending on the specific mutational site of the *TP53* gene.

The finding that the efficacy of platinum‐doublet chemotherapy in first‐line treatment was lower in the late‐onset group than that in the early‐onset group with *TP53* mutations was unexpected (Figure 4C). Therefore, we further compared the overall survival (OS) between the two groups. The median OS was significantly lower in the early‐onset group than that in the late‐onset group (17.8 months vs. 31.2 months, *p* < 0.001, Figure S2A). Additionally, we evaluated the survival period after the failure of first‐line chemotherapy in both groups, which also showed significantly poorer outcomes in the early‐onset group (Figure S2B).

**FIGURE 5:**
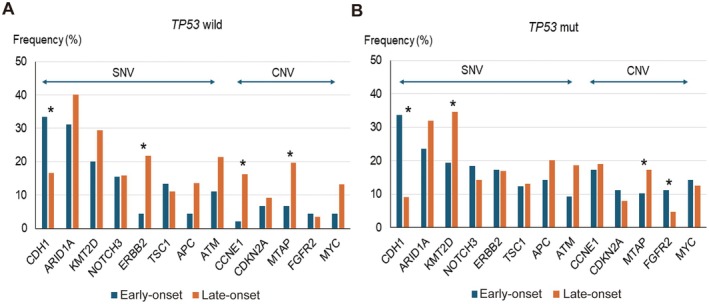
Frequency of variants in genes other than *TP53* in *TP53* wild and mutant groups. (A) Without‐*TP53* mutation (*TP53* wild) group. (B) With‐*TP53* mutation (*TP*53 mutant) group. Bar charts represent the frequency of each variant in early‐ and late‐onset groups. The differences of each variant in early‐ and late‐onset groups are similar between *TP53* wild and mutant groups other than *ERBB2*, *CCNE1*, and *FGFR2*. CNV, copy number variant; mut, mutant; SNV, single nucleotide variant.

To consider the potential impact of genetic variants other than *TP53* mutations, we examined the frequency of each variant in the *TP53* wild‐type and *TP53* mutant groups (Figure 5A,B). The differences in variant frequencies between the early‐ and late‐onset groups showed similar trends in both the *TP53* wild‐type and *TP53* mutant groups, with the exception of a few variants (*ERBB2*, *CCNE1*, and *FGFR2*). Moreover, the frequency of these variants is relatively low. Therefore, we believe that the impact of variants other than *TP53* is minimal. These results suggested that *TP53* mutations play a significant role in influencing the clinical outcomes of GC.

### 3.5 Differential *TP53* Mutation Sites Determine the Sensitivity to the Platinum‐Based Drug in GC Cells

**FIGURE 6:**
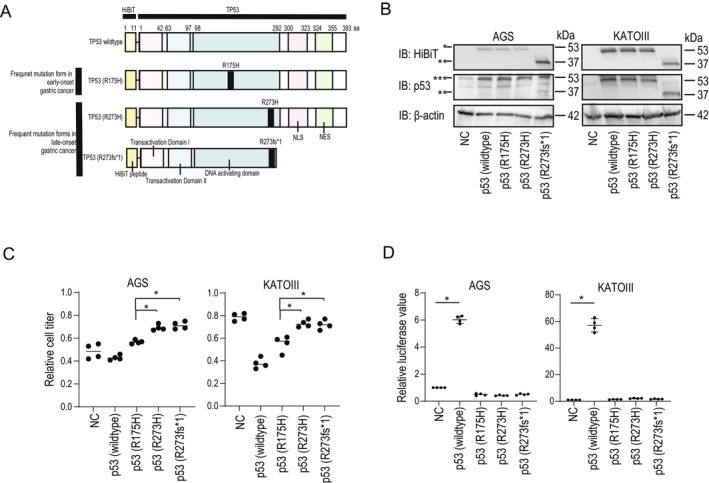
Distinct mutations in *TP53* result in a distinct sensitivity to oxaliplatin. (A) Constructs expressing p53 variants used in this study. All constructs were fused with N‐terminally HiBiT‐tagged to confirm their expression. p53 with R175H mutation was prepared as a representative variant observed in the early‐onset group. p53 with R273H and *TP53* with R273fs*1 were prepared as representative variants observed in the later‐onset group. Wildtype p53 was used as a control. (B) Establishment of AGS and KATO III cells expressing each of the p53 variants. HiBiT tag was visualized by the HiBiT blotting using the cell lysates, in which each of the p53 variants was stably expressed (upper panels). The same membrane was reblotted with p53 antibody (middle panels) and β‐actin antibody (lower panels). IB, immunoblotting. *, HiBiT‐p53 (wildtype, R175H and R273H). **, HiBiT‐p53 (R273fs*1). ***, endogenous p53 (wildtype p53 in AGS cells and null p53 in KATOIII cells) plus HiBiT‐p53 (wildtype, R175H and R273H). Representative images from three independent blotting experiments are shown. (C) Sensitivities of the cells expressing each *TP53* variant to oxaliplatin in vitro were tested. Cell viabilities were determined 3 days after the oxaliplatin treatment (10 μM). Values from the control without oxaliplatin treatment were adjusted to 1, and relative cell titers are shown. Data represent means ± standard deviation (SD) (*n =* 4). *p‐value < 0.05. All statistical analyses were performed using Welch's *t*‐tests. (D) Functions of p53 variants were determined by the reporter assay. Luciferase reporter with p53 responsive elements in its promoter was cotransfected with various kinds of p53 variant‐expressing constructs in AGS (p53 wildtype) and KATO III (p53 null) gastric cancer cells. Relative luciferase activities were determined at 48 h after transfection. Values from cells transfected with a control plasmid were adjusted as 1. Data represent means ± SD (*n =* 4). *p‐value < 0.05. All statistical analyses were performed using the Welch's *t*‐tests.

Based on the above results, we hypothesized that p53 variants frequently observed in early‐ and late‐onset GCs may have distinct functional abilities, leading to different clinical outcomes. To test these possibilities, we examined the functional differences between distinct *TP53* mutations in GC cells by expressing each p53 variant protein (Figure 6A). Sensitivity to oxaliplatin, which is the key drug in GC treatment, differed in GC cells that stably express different variants of p53 mutational forms. Indeed, cells expressing *TP53* with the R175H mutation, which is frequently observed in early‐onset GC, were more sensitive to oxaliplatin than cells expressing *TP53* with the R273 mutation, which is observed more frequently in late‐onset GC (Figure 6B,C). However, mechanistically, the functions of *TP53* as a transcription factor were abolished in all types of variants, as determined using a reporter assay driven by p53 responsive elements. In AGS cells harboring wild‐type *TP53*, forced expression of mutant p53 proteins functioned as dominant negatives. In KATO III cells, which harbor null *TP53*, all types of p53 variants had no effect on the p53 responsive reporter, while wild‐type *TP53* strongly worked on it (Figure 6D). These results suggested that different mutational variants of p53 have distinct functions in a nontranscriptional ability‐dependent manner, leading to different chemosensitivities to platinum‐based drugs in GC cells. These findings may also define different TTF in cases of early‐onset and late‐onset GC with *TP53* mutations.

## 4 Discussion

In the present study, we explored the genomic profiles of early‐ and late‐onset GC using a large‐scale nationwide database of cancer genomics and therapeutics. Although our findings identified the *TP53* mutation as the most frequent alteration in both groups, the specific mutational sites of *TP53* differed between early‐ and late‐onset GC. Furthermore, the different mutation sites of *TP53* potentially influence differential sensitivity to platinum‐based combination chemotherapy.

Genetic mechanisms underlying early‐onset GC remain poorly understood. Although some studies have investigated genomic differences between early‐ and late‐onset GC, these have been limited in scale, and many aspects, such as specific genetic mutations associated with early‐ and late‐onset GC, the pathways involved in their progression, and the impact of genetic variations on treatment outcomes, are unclear [15, 16]. Given the differences in clinical presentations, such as drug sensitivity and prognosis, between these patient groups, we leveraged a large‐scale database of cancer panel results for a comprehensive comparison.

Our analysis revealed that although *TP53* mutations were prevalent in both the early‐ and late‐onset groups, some genes with mutations varied significantly. Specifically, *CDH1*, *ARID1A*, *NOTCH3*, and *TSC1* mutations were more frequent in early‐onset patients, whereas *ARID1A*, *KMT2D*, *APC*, and *ATM* mutations were more common in late‐onset patients, which is consistent with previous reports [17, 18, 19]. These findings highlight the distinct genetic landscapes and suggest that different genetic mechanisms may underlie the timing of cancer onset and contribute to differences in prognosis.

An important finding of this study was the variation in *TP53* mutation sites between the early‐ and late‐onset groups. Previous studies have shown that these variations may affect p53 function. For example, mutations such as R175H, which are common in patients with early‐onset GC, induce genomic instability, leading to favorable responses to therapies, whereas R273 mutations, which are common in patients with late‐onset, are associated with increased malignancy and reduced drug sensitivity [20, 21, 22]. Consistent with these reports, our in vitro results also showed that cells harboring R175H mutations exhibited higher drug sensitivity than those with R273 mutations, possibly through the nontranscriptional function of p53, which supports the longer TTF observed in early‐onset patients, regardless of the use of combined Tmab or Nivo. Therefore, differences in the *TP53* mutation sites may be more clinically significant than previously thought [23, 24].

Regarding the relationship between *TP53* mutation types and drug sensitivity, the efficacy of Nivo has been previously reported. A study using the GI‐SCREEN database demonstrated that patients with *TP53* wild‐type exhibited better progression‐free survival (PFS) than those with *TP53* mutant‐type. Among *TP53* mutations, frameshift mutations were linked with higher response rates and longer PFS than those linked with transversion mutations. In this study, while R175H and R273 mutations were identified as gain‐of‐function (GOF), no significant differences in drug efficacy were observed between the groups with GOF and non‐GOF mutations [25]. These results are consistent with another report [26] indicating that the efficacy of Nivo in gastric cancer does not differ based on the presence or absence of *TP53* mutations. Although previous analyses focused on Nivo monotherapy, our study assessed the impact of *TP53* mutations on treatment outcomes in patients treated with a platinum‐doublet regimen combined with Nivo as the first‐line therapy. We believe that the differences in treatment efficacy observed between patients with and without *TP53* mutations are likely more influenced by the platinum‐based agents than by Nivo. Additionally, a novel finding of our study is that the effects of *TP53* mutation status on treatment efficacy vary depending on the age of gastric cancer onset.

Interestingly, while early‐onset GC is generally considered to have a poorer prognosis [9, 27] than the late‐onset one, our study found a poorer treatment response in patients with late‐onset GC with *TP53* mutations compared to those with early‐onset GC. This discrepancy may be due to the higher prevalence of *TP53* mutation sites, which are associated with poor sensitivity to oxaliplatin in patients with late‐onset GC. Despite this, the traditionally poorer prognosis of early‐onset cancer suggests that factors beyond *TP53* mutations, such as genetic variants or sensitivity to drugs other than platinum‐based drugs, may also play a role. In this study, OS was significantly lower in the early‐onset group than that in the late‐onset group, with a similar trend observed in the survival period following the failure of first‐line chemotherapy. These findings also suggest that other factors, such as differences in treatment responsiveness after second‐line chemotherapy, may surpass the differences in platinum sensitivity ascribed to *TP53* mutation status in first‐line treatment, contributing to the generally poor prognosis in the early‐onset group. Further investigations are warranted to validate these results.

Although the reasons for the differences in *TP53* mutation sites between patients with early‐ and late‐onset remain unclear, our study suggests that genetic mutations can have different effects depending on age and can have different influences on gene function. These findings have significant clinical implications, as understanding specific mutation sites can enhance treatment response prediction and help identify new therapeutic targets, underscoring the importance of further research on gene mutation sites and their functional impacts.

This study has several limitations. First, the analyses were restricted to a Japanese cohort, and no validation using an independent cohort was conducted. Second, the use of five different panel tests made it difficult to clearly distinguish between germline and somatic mutations in most cases. Third, a relatively large number of cases were excluded due to missing treatment data, potentially introducing selection bias. Even though this was an observational study utilizing the results of a comprehensive cancer genomic database, this study elucidated the distinct genomic profiles of early‐ and late‐onset GC. Distinct *TP53* mutation sites and other genetic alterations highlight potential age‐related differences in GC biology. The association between specific *TP53* mutation sites and treatment responses underscores the importance of personalized medical approaches for managing GC. Further research focusing on such genetic markers could facilitate the understanding of the pathogenesis of age‐related GC and the development of tailored therapeutic strategies to improve patient outcomes.

## Author Contributions

**Tomohiro Kamio:** writing – original draft, investigation. **Yoshiyasu Kono:** conceptualization, investigation, writing – original draft, data curation. **Kensuke Hirosuna:** validation, data curation, investigation. **Toshiki Ozato:** data curation, investigation. **Hideki Yamamoto:** investigation, data curation. **Akira Hirasawa:** investigation, data curation, supervision. **Daisuke Ennishi:** investigation, data curation. **Shuta Tomida:** investigation, data curation. **Shinichi Toyooka:** investigation, data curation, supervision. **Motoyuki Otsuka:** conceptualization, investigation, funding acquisition, writing – review and editing, data curation.

## Ethics Statement

This study adhered to the Declaration of Helsinki and was approved by the Ethics Committee of our institution (approval number: 2111‐047) and the Review Board of C‐CAT (C‐CAT Control Number: CDU2022‐012E02). All patients enrolled in the Portal have given written informed consent permitting the secondary use of their clinical data and CGP results for research purposes.

## Conflicts of Interest

The authors declare no conflicts of interest.

## Supporting information


Figure S1

Figure S2

Data S1

## Data Availability

All data are available from the corresponding author upon reasonable request.
