# Supplementary figures and images for: Genomic Differences and Distinct TP53 Mutation Site‐Linked Chemosensitivity in Early‐ and Late‐Onset Gastric Cancer
Source: Cancer Med. 2025 Apr 18;14(8):e70793. doi: 10.1002/cam4.70793 (PMC12007182; doi:10.1002/cam4.70793)

Supplementary Figure 1

A

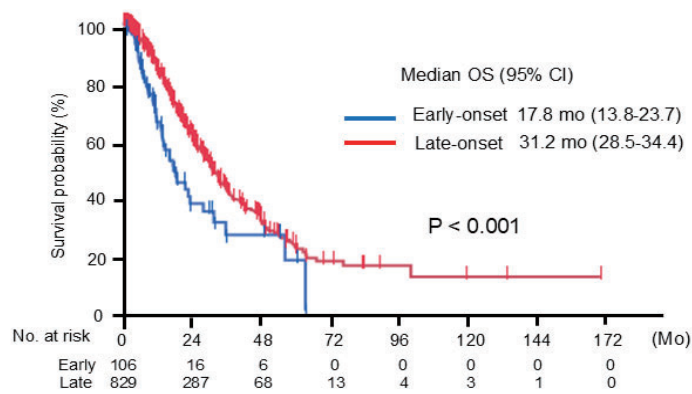

B

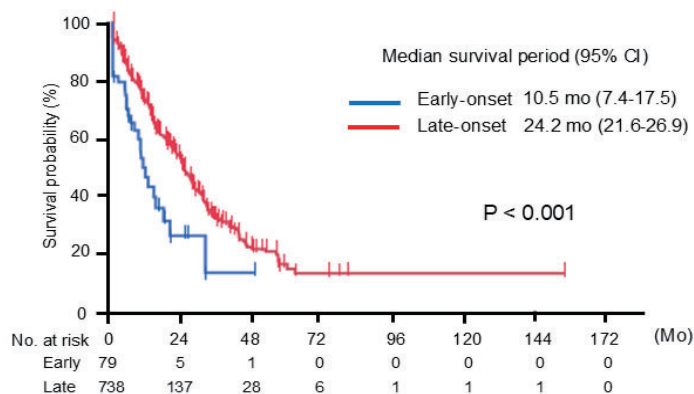

Supplement: Supplementary file 1 — Figure S1 [file CAM4-14-e70793-s003.pdf]

Supplementary Figure 2

A

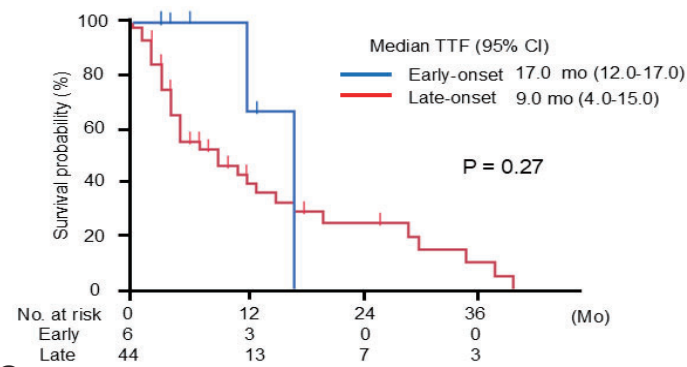

B

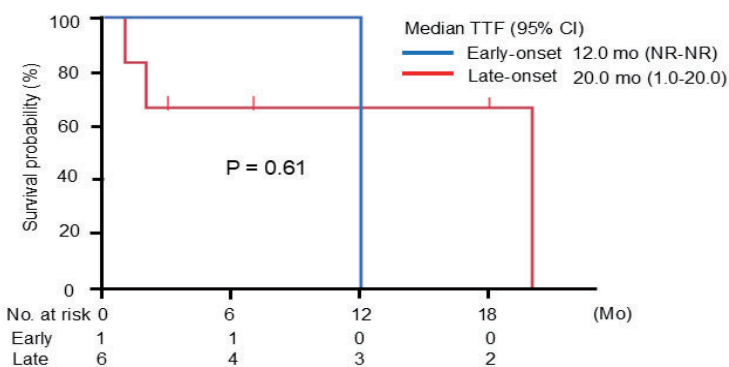

C

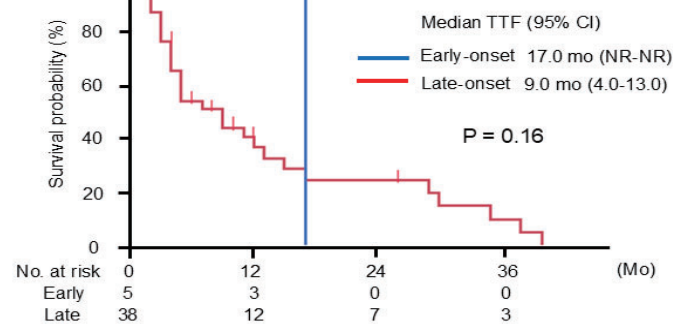

D

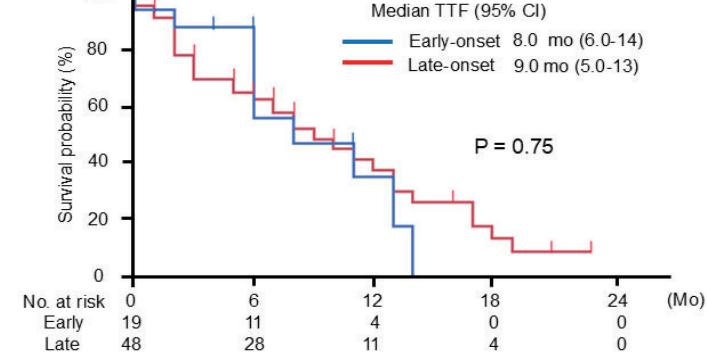

E

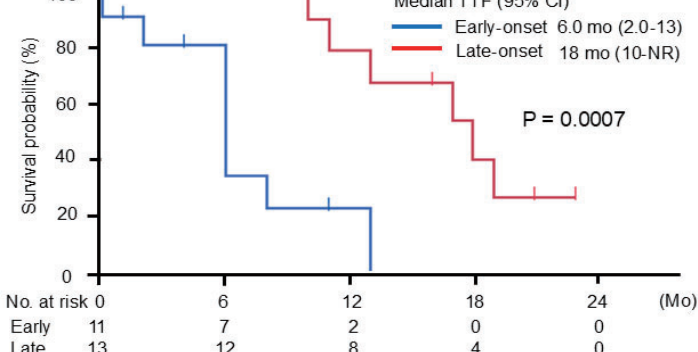

F

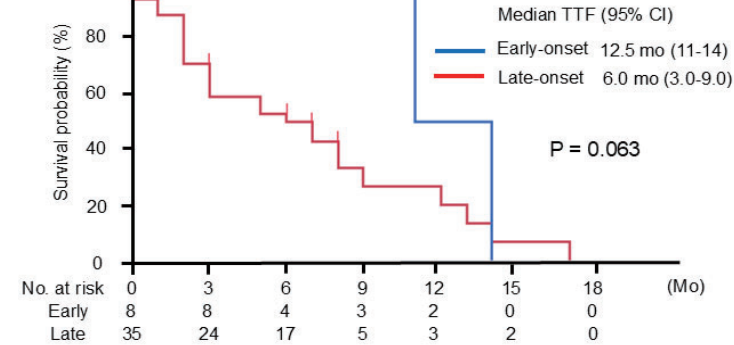

Supplement: Supplementary file 2 — Figure S2 [file CAM4-14-e70793-s002.pdf]
