# Supplementary material for: Genomic Differences and Distinct TP53 Mutation Site‐Linked Chemosensitivity in Early‐ and Late‐Onset Gastric Cancer
Source: Cancer Med. 2025 Apr 18;14(8):e70793. doi: 10.1002/cam4.70793 (PMC12007182; doi:10.1002/cam4.70793)
Supplement: Supplementary file 3 — Data S1 [file CAM4-14-e70793-s001.docx]

**Supplementary Information**

**Genomic differences and distinct *TP53* mutation site-linked chemosensitivity**

**in early- and late-onset gastric cancer**

Tomohiro Kamio, Yoshiyasu Kono, Kensuke Hirosuna, Toshiki Ozato, Hideki Yamamoto, Akira Hirasawa, Daisuke Ennishi, Shuta Tomida, Shinichi Toyooka, and Motoyuki Otsuka

**Guide to the Supplementary Information**

**Supplementary Figure Legends**

**Supplementary Figures 1 and 2**

**Supplementary Figure Legends**

**Supplementary Figure 1. Time to treatment failure in early- and late-onset groups conducting the platinum-doublet regimen with trastuzumab or nivolumab as first-line chemotherapy.**

(A) Kaplan-Meier curves for time to treatment failure (TTF) using the platinum-doublet regimen combined with trastuzumab (Tmab) across all early- and late-onset groups. (B) Kaplan-Meier curves for TTF when using the platinum-doublet regimen combined with Tmab in patients with the *TP53* wild group. (C) Kaplan-Meier curves of TTF using the platinum-doublet regimen combined with Tmab in patients with the *TP53* mutation group. (D) Kaplan-Meier curves for TTF using the platinum-doublet regimen combined with nivolumab (Nivo) across early- and late-onset groups. (E) Kaplan-Meier curves for TTF using the platinum-doublet regimen combined with Nivo in patients with the *TP53* wild group. (F) The Kaplan-Meier curves of TTF when using the platinum-doublet regimen combined with Nivo in patients with *TP53* mutations. Despite separate analyses for the Tmab group and Nivo groups, the effectiveness of chemotherapy varied between early- and late-onset cases, depending on *TP53* mutation status. TTF; time to treatment failure, CI; confidence interval, Mo; months, No; number.

**Supplementary Figure 2.** **Overall survival and survival period after the failure of platinum-doublet regimen as first-line chemotherapy in early- and late-onset groups.**

(A) Kaplan-Meier curves of overall survival (OS) in early- and late-onset groups. (B) Kaplan-Meier curves of survival time after the failure of the platinum-doublet regimen as first-line chemotherapy in early- and late-onset groups. Both OS and survival period after the failure of the first-line treatment were poorer in the early-onset group compared to that in the late-onset group. OS; overall survival, Mo; month, CI; confidence interval, No; number.
